# The Republic of Fear: Mental Illness in the Finnish Civil War of 1918

**DOI:** 10.1093/shm/hkac065

**Published:** 2023-02-23

**Authors:** Petteri Pietikainen

**Affiliations:** History of Sciences and Ideas, University of Oulu, P.O. Box 1000, FIN-90014, Oulu, Finland

**Keywords:** civil war, mental illness, psychiatry, fear, Finland

## Abstract

This article examines the links between mental illness and the Finnish Civil War of 1918. Based on the study of patient records from a large state mental hospital, the article discusses the mental wounds of both servicemen and civilians and focuses on fear as an essential component in the onset of mental disorder. An examination of patient records reveals how civil war affected the mental health of ordinary people and created a collective psychological atmosphere of fear and anxiety. What this article also demonstrates is that, during and after the war, patients who were mentally scarred by the atrocities were neither categorised nor diagnosed any differently from other mental patients. By focussing on patient experiences in the ‘mini-society’ of a mental hospital, this article aims to give a nuanced account of the ways in which civil war can affect mental health on both the individual and collective levels.

In recent decades, the Finnish Civil War of 1918 has been studied from many different angles. The chain of events leading up to the war are now well-known, the war itself is meticulously researched, and new approaches to the conflict include memory studies, gender history, psychohistory and the cultural history of war.^1^ We know the number of deaths, post-war prisoners, orphans and widows. What remain largely unexplored are the effects of the Civil War on the mental health of Finns. Based on the study of patient records from a large state mental hospital, this article examines the links between the Finnish Civil War and mental illness. My focus is on the ways in which both servicemen and civilians were—to use the idiom from the twenty first century—*traumatised* by the war.

When looking at studies of civil war, it seems appropriate to cite Leo Tolstoy, whose *Anna Karenina* begins with this famous sentence: ‘All happy families are alike; each unhappy family is unhappy in its own way’. To apply this idea to civil wars, one could say that all wars between nation states and other political units are alike, whereas each internal armed conflict is internal in its own way.^2^ This is of course an exaggeration, but I would argue that, when a historian studies a particular civil war, to compare and contrast it to other civil wars in history is of very limited use, even less so than in the case of armed conflicts between sovereign states. This is because each civil war is so deeply embedded in a particular political context which has its own history of social structures, political culture, economy and institutions (not to mention religions and intellectual currents), that one is forced to study something that is, *sui generis*, one of a kind.^3^

At the same time, civil wars all obviously have a supranational context. In the Finnish Civil War, this context was World War I and its ramifications—especially the February and October Revolutions in Russia, and the subsequent disintegration of the Russian Empire (at the time of the October Revolution, Finland was still part of the Empire as an autonomous Grand Duchy). The successful Bolshevik takeover in October 1917 (7 November in the Gregorian calendar) encouraged the more radical Finnish socialists within the Social Democratic Party to consider an armed uprising of their own. Thus, the Finnish Civil War should be placed in the context of World War I, as one of the upheavals it brought about in the European fringe areas, including the more peripheral parts of the Habsburg Empire, the Baltic region and the Russian Empire.^4^

Civil war is an exceptionally shattering experience to the divided nation—the whole social, moral and psychological fabric of the nation is more or less torn apart. And the Finnish Civil War was a short but very violent conflict, affecting most Finns and especially those living in the areas taken over by the socialist Red Guards—not so much because the Reds were dangerous to the local population (sometimes they were), but because there were supporters of both the Reds and anti-socialist Whites living in these areas, in the same towns, villages and neigbourhoods.^5^ Many Finns, worried and frightened at the escalation of violence, supported neither side nor wanted to take sides, at least in public.^6^

Regarding mental suffering in the Finnish Civil War, a general observation to make at the outset is that, in recent decades, research on the psychiatric casualties of both the ‘Great War’ and World War II has greatly expanded. There are now numerous historical studies on war psychiatry, which explore and discuss various aspects of ‘shell shock’, ‘war neurosis’, ‘male hysteria’, ‘combat fatigue’ and other designations for war-related psychological scars.^7^ These studies have deepened our understanding of the effects of war on the minds of soldiers, and to a lesser extent, of civilians, and we have a fairly comprehensive picture of the roles and functions of twentieth-century war psychiatry. Compared to the two world wars, there is very little historical research on mental disorders and psychiatry in civil wars of the modern era.^8^

From the scholarly perspective, what is noteworthy in the Finnish Civil War is that the patients who were mentally wounded by the war were neither categorised nor diagnosed any differently from other mental patients. They were treated in the same way as all other mental patients, the only difference being that the reason for their admission was the Civil War. The two probable reasons why there were no particular diagnoses related to war patients was, first, that Finland did not participate in the Great War, and so the psychiatrists had no experience of war-related traumas when the Civil War broke out. Second, the war was both a sudden and relatively short conflict (little more than 3 months), and for this reason mental hospitals were quite unprepared to function as military hospitals and to provide care to acute psychiatric patients. In the absence of ‘war psychiatry’, there were not any specific diagnoses, concepts or theories to distinguish ‘war patients’ from ‘civilian patients’.

The principal source material used in this article are 24 patient records from Pitkäniemi, the state mental hospital that provided care to psychiatric war patients in 1918. I read all patient records from 1918 and noticed that, while the war affected almost every patient’s life at least until the end of fighting in May, these 24 patients were admitted to the hospital because the war had seriously affected their mental health. As a hospital, Pitkäniemi was the natural choice for this historical inquiry, because during the war it was located in the midst of the large southwestern area of Tavastia controlled by the Reds, which meant that the tensions between the Red Guards and the White Guards (also called the ‘Protection Guards’) and the civilian population ran high in the regions around the hospital. My presentation of the patient cases will illustrate the ways in which the Civil War affected the mental health of ordinary people living in the Red zone, which was also a war zone.

Theoretically and methodologically, this article combines the social, intellectual and medical history of madness. The social historical dimension should be relevant from the start: the focus is on the effects that civil war had on ordinary people. Meanwhile, the intellectual historical dimension is broadly conceived as addressing the political, religious and cultural convictions—mental habits, as it were—of the patients discussed in this article. Finally, the medical dimension provides an essential *mise en scène* for an inquiry that is set in a mental hospital with medically trained staff, facilities and treatments. While this article certainly moves between the micro-(patients), meso- (Pitkäniemi and other mental hospitals) and macro-levels (politics of war), its main emphasis is on links between the micro and macro: I use the hospital records of individual patients as a window through which to explore how civil war as a social and political phenomenon affected the mental health of ordinary people and created a collective psychological atmosphere of fear and anxiety. In other words, by focussing on patient experiences in the ‘mini-society’ of a mental hospital, this article aims to give a nuanced account of how a civil war affects mental health on both the individual and collective levels.

I will first discuss the key-concept of fear in the context of the Civil War, and then I will look at how the war affected Pitkäniemi Hospital itself. In the following sections, I will examine the patient cases in the thematic framework of fear. From there I move on to discuss the impact of war on other mental hospitals in Finland. I then briefly examine the aftermath of the war, including field court-martials, prison camps, and psychiatric and psychological accounts of the war. On the basis of 24 patient records, plus a few others taken from another mental hospital some years after the war, I cannot make strong generalisations about the characteristics of ‘war traumas’ that are particular to civil wars, but at the end of the article I draw some conclusions about the Finnish Civil War and its impact on the mental health of Finns.

## A Republic of Fear

In his *Theory of Moral Sentiments* (1759), Adam Smith called fear and anxiety the ‘great tormentors of the breast’.^9^ Researchers of fear have tried to reveal how it is, exactly, that we acquire knowledge about these great tormentors?^10^ To approach fear as an object of scientific knowledge is to ask questions about its reality as a bodily reaction, emotion (evolutionary evolved or not) and lived experience. In historical research on fear, the principal question is, to put it simply, what do the sources say about fear—‘say’ in the literal meaning of either being explicit about the sensation (‘I was afraid’), or making implicit references to something that could be interpreted as denoting fear (‘she was anxious about the forest fire near her house’).

Historians of fear in the twentieth century have often looked to wars and other conflicts as source material, as expressions of fear and anxiety are anything but difficult to find there. For example, Jan Plamper has studied changes in the discussion of fear among Russian soldiers from the early nineteenth to early twentieth centuries;^11^ while Michal Shapira—in line with a growing concern over the emotions of civilians—has shown how anxiety and fear began to be studied and managed by British psychoanalysts and other behaviour experts in the 1930s, as ‘fear slowly became a universal, even “natural” reaction to war’.^12^ Joanna Bourke and Ute Frevert have meanwhile argued that, intrinsic to the emotion of fear, are power relations—fear mediates between the individual and social, aligning individuals within their community (this is true of many other emotions as well, especially those involving moral emotions, such as anger, sympathy, shame and remorse).^13^

Common to all the mental patients discussed in this article is the fact that none of them was truly an outsider in the Civil War. Whether they had been civilians or servicemen for either the Reds or the Whites, all had certainly faced some aspect of the horrors of war. Like most British troops in the First World War, these servicemen had, as medical anthropologist W.H.R. Rivers then noted, ‘volunteered or been conscripted into the army and trained in great haste’, and as a consequence ‘they had not had the time to build up an effective mechanism to deal with fear and anxiety’.^14^ When Finnish hospital psychiatrists referred to fear in the patient records, they were quite explicit about this emotion: they either recorded what the patients themselves said, or they made their own observations about the mental (and physical) condition of their patients—their fear, anxiety and/or sheer terror clearly manifest in each patient’s behaviour and outlook.

In the patient records there is, however, a curious absence of any psychological or physiological interpretations of fear. Although Finnish psychiatrists in 1918 were closely following developments in (especially German) psychiatry and psychology, when writing about these patients, they did *not* refer to ‘degeneration’, ‘the constitution’, ‘the unconscious’, nor any of the other psychomedical concepts that were circulating around this time.^15^ Neither did they apply moral categories in their assessment of their patients’ condition: the patients were not called cowards, soft or lacking in resolution or toughness. Whereas Michal Shapira has noted how, during the Great War and its aftermath a ‘display of fear among male soldiers [in Britain] was still stigmatised and seen as a symptom of cowardice’,^16^ I have not found a similar ‘discourse of cowardice’ in accounts of the Finnish Civil War. I would argue that an important reason for the absence of moral blame in the patient records or professional publications is that Finnish psychiatrists readily understood and, in many cases, possibly empathised with their patients’ mental anguish. Pitkäniemi Hospital—the principal *mise-en-scène* of this article—was located in an area controlled by the Reds, and by simply being doctors, psychiatrists would have been seen as bourgeois class enemies of the insurgents. Although the revolutionaries did not interfere with the daily activities of the hospital, the medical staff would have been clearly aware of this relatively precarious position. Although they almost certainly opposed the Reds and their political goals, they refrained from using derogatory language in patient records referring to Reds or those sympathetic to the insurrection or socialism; nor was such language used in records even after the war, when many of those suspected of being sympathetic to the Red cause were being incarcerated in large prison camps and put on trial.

For Finns caught up in the violent cauldron of the Civil War, to be afraid was a natural emotional reaction not only to the atrocities themselves, but to the overall atmosphere of hate and animosity that created a collective mentality of distrust and poisoned the fabric of Finnish society in a way that had never happened before. Finland had no army of its own, and it had not participated in the Great War so, unlike many other parts of Europe, there were no restless war veterans returning home. In fact, the nation had not experienced a war since the early nineteenth century, and there was no culture of militarism.^17^ No wonder then that, at least before the violent week of general strike in November 1917, nobody could foresee the abyss into which the country was about to plunge.^18^

At Pitkäniemi in 1918, psychiatrists recognised that many of their new patients were mentally unwell because of the war, and they paid attention to the experience of fear as an essential factor associated with the onset of mental illness. True, they also emphasised hereditary factors in the sense of paying attention to the question of whether mental disorders run in families,^19^ but this did not necessarily contradict the psychological importance of fear in mental illness. On the contrary, it was quite plausible for psychiatrists to assume that those with a ‘hereditary burden’ (usually meaning that the patient had mentally or ‘nervously’ ill family members) were more inclined to react ‘abnormally’ to events in their environment.^20^ As the number of patients admitted to mental hospitals for war-related reasons remained, after all, quite small, it was quite reasonable for the psychiatrists to think that their frightened patients were exceptional cases. This, of course, could mean that the mental constitution of these patients was, at least to some extent, unusual or abnormal, but it could also mean that the patients had simply gone through some unusually shocking experiences. In 1918, the German psychiatrist and neurologist Karl Kleist, who had studied more than 100 soldiers stationed on the Western Front, coined the term ‘terror psychosis’ (*Schreckpsychose*) to refer to the type of reactive and transient ‘psychogenic’ condition that characterised the suffering of most of the patients discussed in this article.^21^

As the political scientist Corey Robin has pointed out, fear is a ‘highly plastic emotion’, and it does not need to ‘resemble the terrorized face in Edvard Munch’s famous portrait The Scream’. Instead, the marks of fear can look reasonable, purposive and useful, as they did in Cold War America during McCarthyism.^22^ In effect, the only reason this article centres around the concept of fear is that its main actors—mental patients and their relatives—constantly referred to the sensation of fear in their accounts of the onset of symptoms. And the kind of fear they talked about does resemble The Scream rather than the moderate ‘everyday’ forms of fear of the McCarthy era United States.

## Fear and Finnish Folk Beliefs

To better understand the cultural context of ‘being frightened’, it is instructive to look at the studies of traditional folk beliefs among the Finns and the Finno-Ugric peoples in Russia. Ethnologists doing field work among these peoples in the early decades of the twentieth century recorded the belief that, if you suddenly become frightened, you may ‘go crazy’. Until well into the twentieth century, most Finns usually employed traditional colloquial terms, such as ‘madness’ (*hulluus*) and ‘go crazy’ when they discussed mental disorders, or when they referred to people who behaved in a strange way.

According to folk beliefs, there were certain places and situations that could trigger fear and fright, including fires, storms, graveyards and sites where people had died, as well as supernatural spirits in the woods, lakes and wind. Women were particularly vulnerable to fright during pregnancy, which made them sensitive and exposed to risk. To become frightened was to risk losing the immaterial essence of one’s being, or *haltia*—a near-equivalent to the Christian concept of soul. When one was in the vulnerable state of fright, *haltia* could also be taken by spirits, which could then cause physical or mental illness. Those who had lost their *haltia* needed to get it back, or they were in danger of losing their mind for good. Madness also threatened those invaded by an alien spirit, and it could manifest itself in epileptic seizures or the state of enrapture. In such cases, the malicious spirit had to be driven out of the body by frightening the victim. Thus, fright seemed to have a double function in the Finnish folk belief system—it could make a person ill, but it could also heal.^23^

In 1918, such folk beliefs were still alive in Finland, especially in the rural areas where traditional customs and beliefs lived side by side with more modern technology, health care, education, commerce etc. Doctors generally discounted what they saw as old wives tales’ and regarded folk healing as quackery, but they also knew that, in rural areas, locals sometimes sought the help of folk healers, and that ‘common people’ believed that either supernatural entities or other people, such as malevolent neighbours, could use magic, incantations and other supernatural tricks to drive their enemies crazy, make them have nightmares or upset them. To some extent, traces of these folk beliefs can be seen in the post-Civil War ‘myth of madness’ that was created by the Reds and their supporters when they learned that the White government would not put the perpetrators of the ‘White terror’ on trial to face charges. Disappointed, they created stories to console themselves about the White ‘butchers’ coming to an unhappy end through madness, suicide, alcoholism, nervous breakdown or a fatal illness.^24^ For those Reds who were religious, to have faith in some sort of retribution was particularly acute, because the Lutheran State Church had been firmly on the side of the Whites.^25^

That the war was truly a civil war was a surprise to many of the Whites, who initially thought they would be fighting against the Russians. To some extent, this also applied to the Reds, who thought they were fighting against capitalist exploiters and the bourgeoisie. In his war diary, the young Red Guard, Viljo Sohkanen, expressed the genuine surprise that he and his comrades felt when, after an early battle with the White Army, they noticed that the dead Whites were ordinary workers with calluses on their hands and the same shabby clothes as themselves. ‘Where are our real enemies that we were supposed to fight against?’, Sohkanen asked. ‘Something must be wrong, very wrong […] Who’s being deceived now?’^26^ When it dawned upon these amateur soldiers that they were really fighting against their fellow Finns, and people like themselves, it must have been a sobering discovery. When this fact sank in, it inevitably heightened social and psychological tensions, especially in the areas controlled by the Reds, such as Tavastia, where the Pitkäniemi Hospital was located.

## Pitkäniemi State Mental Hospital in the Civil War

When the Civil War broke out in late January 1918, the Red Guards took control of the southern, most developed part of Finland. This was the region where the political and economic tensions had accumulated over the past 20 years as industrialisation had created a new kind of urban, politically organised and class-conscious working class that voted for the Social Democrats and aimed for political and social reforms. In the more agrarian and less developed parts of the country in the north and east, the anti-socialist White Guards, which by then had officially become government troops, managed to gain control, sometimes after fighting with the local Red Guards. Helsinki, the capital of Finland, was firmly in the hands of the Reds, and so were the other major towns in the south, including Tampere, an industrial centre and an important Red stronghold. The modern state mental hospital of Pitkäniemi, established in 1900, was located in the municipality of Nokia, close to Tampere. With close to 500 beds, Pitkäniemi admitted patients from the area that was now controlled by the Reds. During the war, there were also several Red Cross hospitals in Tampere.

When the Red Guards arrived in Pitkäniemi, they confiscated weapons found on the premises (e.g. in the house of the chief physician, Ernst Therman). A group of them then remained in the hospital to ensure there was no underground communication between medical staff and the Whites. There was a good reason to take such a precaution, because the overwhelming majority of doctors supported the White Army, and in some hospitals they were hiding weapons and communicating in secret with local Whites. In Pitkäniemi, the Red Guards also controlled traffic to and from the hospital and patrolled the area, but they did not intervene in the daily activities of the institution.^27^

During the course of 1918, Pitkäniemi admitted 24 patients, whose patient records revealed their cause of illness to be the ‘political situation’, ‘tumultuous times’ or the ‘Red rebellion’. Of these patients, 21 were men, five were Whites, another five were Reds and the rest I would categorise as being more or less innocent bystanders. They were all given diagnoses that indicated a serious mental disorder: 10 were diagnosed with *dementia praecox* (this diagnosis was replaced by schizophrenia in 1921 in Finland), and 13 with manic-depressive insanity. One male patient was given the dual diagnosis of *imbecillitas* and mania. These psychiatric diagnoses, especially those of Emil Kraepelin—including *dementia praecox* and manic-depressive insanity—were imported from Germany, where Finnish psychiatrists made study trips in the early decades of the century. As a consequence, psychiatric theories and therapies from Germanophone Europe (Germany, Austria, Switzerland) exerted a strong influence on Finnish psychiatry.^28^ Although on the scientific periphery, Finland thus remained connected to European academic centres.

According to the Board of Medicine’s Statistical Yearbook for 1918, state mental institutions admitted a total of 122 patients whose illness was ‘assumed to be caused’ by the ‘war situation’ (89 patients), or by the dual reasons of ‘heredity and the war situation’ (33 patients). Of these patients, 92 were men and 30 women.^29^ The real number of patients admitted for war-related reasons must have been higher though, because the war is most likely to have also affected those patients whose illness was attributed to causes such as ‘heredity’, ‘shock’ (if patients became acutely frightened of some particular thing), ‘imprisonment’ or ‘unknown reasons’. This last category of supposed causes was by far the largest one: the cause of illness for 234 patients was noted as ‘unknown’.^30^

In the following sections, I present 12 of these war patients, or exactly half of the 24 officially recorded ‘civil war cases’ at Pitkäniemi. I chose them for the following reasons: (i) their experiences represent different phases and aspects of the war; (ii) in their records, the circumstances around the onset of mental illness are described in a more detailed fashion than they were for the other half of the war patients; and (iii) the accounts of their experiences illustrate various aspects of becoming affected by a civil war.

## War Patients in Pitkäniemi Hospital^31^

As soon as open conflict broke out between the Reds and Whites at the end of January 1918, newspapers on both sides began to tell stories and spread rumours about killings, tortures and executions committed by the other side. Usually, the stories about torture were fictional, but they fanned the spirit of revenge and mutual hatred.^32^ This meant that within only 2 months of gaining independence, the Republic of Finland had become a republic of fear. What the following cases highlight is how the experience of fear is important in understanding war-related mental disturbances.

### Fear of death.

In April, Kaarlo J., a 31-year-old rural police chief, was admitted to Pitkäniemi. His patient file includes a statement from his mother, who wrote that her son’s illness had been triggered by a dramatic chase a few days earlier. As police chief in a rural municipality in Tavastia, Kaarlo had been a natural target for the Reds, who arrived in the village one day and tried to capture him. Chased by the Reds, Kaarlo escaped across forests and over ice, and, after 2 days, arrived in Tampere, where his parents lived. Exhausted and frightened, he kept dreading that the Reds would come and do away with him—even though, by this time, Tampere was controlled by the Whites. At home, Kaarlo was slightly confused, and then he became sleepless and restless. One day, he tried to cut his throat with scissors, but was stopped and taken to a doctor who referred him to Pitkäniemi.

In the ward, Kaarlo was quiet and suspicious. He saw shadowy ghosts and said he felt like his whole body was charged with electricity. He was still terrified that ‘they would take his life’, thinking that the hospital was controlled by the Reds, then gradually he calmed down. One day in May, he left the hospital without permission and went into the local village to buy cigarettes. When he returned, he started to display symptoms of insanity again, which now included a fear of being seen as an informer for the Reds. His condition deteriorated over the summer: he shut himself off from the world, and in this ‘stuporous’ state, no one could make contact with him. Diagnosed with *dementia praecox*, Kaarlo was discharged in September with his condition ‘unimproved’. There is no mention in his patient file about where he was then taken.^33^

### Fear and religion.

In the early twentieth century, ordinary Finns equated psychology with the soul—seen as an immaterial, metaphysical entity separate from the body, especially for the majority of the population who remained Christian. Finns typically believed there were ‘sicknesses of the soul’, and these sicknesses referred either to the religious sphere (despair over one’s salvation, for instance) or mental illnesses proper. Most people whose distress was not related to religious musings or spiritual despair tried hard to avoid the stigma of mental illness; they might say something like ‘no I’m not crazy, I’m only nervous because I slept badly, and I’ve got headaches and pains in my heart (or, in the stomach, back, limbs, etc.).’ From the patient records of mental hospitals, we can see just how much religion still held the minds of many in its thrall in early twentieth-century Finland.^34^ Many Reds, especially the women, were religious, though they rightly saw the Lutheran Church as an ally of the Whites.^35^

I will start with Anna T.,^36^ a 47-year-old wife of a tailor and mother of four children, who was taken to Pitkäniemi in mid-February. Anna was in a state of shock after a violent incident in her village: on January 31, a group of White Guards from Tampere had surrendered to the Reds after a fight. What happened next was to set the tone for the war: another group of Red Guards, sent from Tampere, arrived on the scene and, without any warning, started to shoot the Whites, who had lined up in front of a horse stable. When the mindless shooting was over, 15 Whites were dead, including nine who were students at Tampere Polytechnic Institute. In addition, 28 Whites were wounded. News about this brutal execution at the beginning of the war sent shock waves across the country, and the vengeful White Guards made it their policy not to take prisoners. This in turn incited the Reds to act in the same merciless manner, though much depended on who was in charge of the troops when enemies were captured. This would be one of the first in a series of impromptu executions that came to characterise the Civil War.^37^

In early 1917, Anna had begun to attend meetings of the local workers’ association, and, according to her brother-in-law, she had been somewhat ‘agitated’ after these meetings. In early 1918, she became fearful and began to suspect that the ongoing ‘revolution’ might end badly for the socialists. Her talk often became confused, she had trouble sleeping, and—worst of all for her family—she started to fear poisoning. She was then taken to a local doctor, who issued her with a certificate which stated that she was having delusions about people hating her.

In a psychiatric interview in Pitkäniemi, Anna sat in bed making ‘theatrical gestures’, saying that capitalists would kill all socialists, including women and children. What exacerbated her condition was the religious anxiety she felt about her soul: ‘I have lost my religion, I can never get it back’. In the ward, she spent most of the time silent. After the Christmas of 1919, she was released from hospital with her condition ‘unimproved’ and a diagnosis of *dementia praecox* (followed by a question mark).^38^

Another female patient who was terrified by violence turned to the Bible to make the horror of war less painful. Erika R. was a 29-year-old shop assistant who was taken to Pitkäniemi in February 1918. Her illness was triggered by a battle, which was fought near her home earlier the same month, and in which her house got hit by stray bullets. Shocked by the war right outside her door, Erika began to read the Bible. Gradually, she became totally silent, except when she was reading the Holy Book. Occasionally, she would stand up and proclaim that the new day was dawning. Believing herself to be persecuted, she kept on reading the Bible feverishly. One day she told her family that the Holy Ghost was bestowed upon her, and the following day she said she was going to the ‘Wedding of the Lamb’ (i.e. Christ). She ran away to a neighbour’s, where she stayed until taken to the hospital.

In Pitkäniemi, Erika was at first totally confused, screaming and moving around restlessly. Then she calmed down, and became focussed and responsive, her mood was elevated, and she started to work. In early September, after little more than 6 months in the hospital, she was discharged as fully recovered and diagnosed with having suffered manic-depressive insanity.^39^

A few days after Erika, Anton H., the 29-year-old son of a farmer, was admitted to Pitkäniemi. His father, who had escorted him to the hospital, told the psychiatrist on duty that Anton’s mother was ‘nervously ill’ and that his son had been weak in body when he was young. For some time now, he had been in charge of the family farmhouse, but a week previously, Anton heard that the Red Guards had murdered his two second cousins and captured his two brothers, and nobody knew what had happened to them. Soon after this shocking news, Anton’s mental health began to deteriorate; he became fearful and ran away to the village, refusing to return to the farm. Since then, he had been restless, threatening and occasionally violent; he also admitted having seen apparitions. The local doctor reported in the medical certificate that ‘Anton violently opposes examination, clenches his fists and cannot tolerate his father. He is agitated and seems to hate the “Reds”’.

Upon admission to Pitkäniemi, Anton kept on repeating the same religious terms for a day and a night. There are no further references to religion in his record, but it is quite evident that, just like Anna and Erika, Anton was either trying to make sense of his agony with the help of religion or was worried about his soul. After a month, he complained about feeling restless and unhappy, and that he wanted to go to another ward to see if it would improve his condition; occasionally, he burst into tears. In June, he was reported as alternating between agitation and apathy. The following month, Anton was discharged as ‘improved’ with the diagnosis of ‘dementia praecox’ (followed by a question mark).^40^

As in most patient files of the time, there was no mention of any specific treatment administered to Anna, Erika or Anton. During this era, agitated mental patients were usually given prolonged baths, drugs (e.g. barbiturates as sleeping medication), and what in the 1930s came to be called ‘work therapy’: patients were encouraged to do something useful, which in the case of female patients mostly meant cleaning, washing, knitting, weaving and helping in the kitchen or the laundry. Male patients in turn worked in the fields, stables and gardens. An ability and willingness to work indicated that, sooner or later, the patient might be able to return to the community, if not entirely capable of making an independent living, then at least as a much lighter burden to society. In Finnish mental hospitals, work was seen as a natural stepping stone towards (and justification for) the eventual discharge of the patient from the hospital.^41^

### Fear of becoming involved in the war.

The 25-year-old son of a farmer Eino M. was taken to the hospital in early June. According to his father, Eino became frightened in mid-March, when a Red patrol came to his house to demand horses. He had a good reason to be scared: the Reds had killed his brother. After the Reds left the house, Eino became fearful and sleepless, burst into tears often and did not want to eat. He hallucinated, became paranoid and started to read the Bible. He also appeared to harbour suicidal thoughts, so his worried family took him to a private sanatorium, but when his condition became worse there, they decided to take him to Pitkäniemi.

In the ward, Eino was sad, tense and dispirited. He knew where he was, and he admitted feeling ‘nervous’; ‘I reckon it’s mental illness. I got so scared when the Reds stood outside the door of the sauna and in an angry voice told me to harness my horses to the cart. Then I started to feel cold and my thoughts became confused.’ In the autumn, Eino was restless and impatient, and wanted to go home; occasionally, he had to be spoon-fed. By January 1919, he was spending his days sitting in one place and gazing at the ground. In the early summer, he was released from hospital at his father’s request. Obviously, the medical staff at Pitkäniemi did not have much confidence in Eino’s recovery, as his condition was marked ‘unimproved’ and he was given the more pessimistic diagnosis of *dementia praecox*.^42^

Another patient frightened by the Reds was Kustaa L., a 41-year-old farmhand and father of three. In April 1918, he was the only man in a house from which the others had escaped. One day a Red patrol arrived and started to plunder the house, but Kustaa managed to escape into the woods, where he stayed the whole day. Afterwards, he started to mutter sentences to himself like ‘sure enough, the Whites will be coming soon too’. He had mood swings, and it became increasingly difficult for him to work—at times he was suspicious, anxious and irritable, then at other times cheerful.

Kustaa was taken to Pitkäniemi in mid-June. In the ward, he was calm and aware of his surroundings. He said that he had been short-tempered and violent at times (he had broken windows), but he could not explain why—he still had moments when he felt ‘out of control’ and ‘dizzy, as if there were bad fumes in my head.’ In late June, he admitted having auditory hallucinations, and explained he suffered from a ‘spiritual disease’, not any mental illness. Later in the summer, his behaviour became more organised and he was willing to work. In August, Kustaa was discharged in full health, diagnosed with having suffered from manic-depressive insanity.^43^

In the next two cases, the immediate cause of illness was the fear of being conscripted into the Red Guards. In June 1918, Aleksanteri K., a 25-year-old carpenter and the son of a tenant farmer was admitted to Pitkäniemi. According to the anamnestic information, Aleksanteri had been in full health, and known as a cheerful person, diligent worker and excellent skier. In February, a Red patrol came to his house and forced him and one of his brothers to join the Red Guards. After this incident, his near ones noticed he had become melancholy and sleepless. Wondering whether it was right to join the Red Guards, he stopped working and sought solitude. Thereafter, he became fearful of the Reds: he was restless, thought he heard shooting, and imagined them lurking under the bed, behind doors and virtually everywhere. He also refused to take any medication as he was afraid of getting poisoned. According to one anonymous source—most likely a family member—he was also having trouble in love, as he often talked about a girl who was ‘no longer promised to him’.

In the ward, Aleksanteri was disoriented and talked to himself. He admitted being afraid of the Red Guards and thought the food was poisoned. After this admission, he became unresponsive and started to cry. He remained in this condition for months. In January 1919, he spent days doing nothing, except sitting on a bench and eating reluctantly. Years went by and, little by little, Aleksanteri’s condition …improved! In November 1924, his father came to take him out of the hospital. By this time, he had a full understanding of his illness. He said that the events surrounding the ‘uprising’ had made such a strong impression on him that he ‘lost his mind’ and became ‘insane’. Before he left the ward, he warmly thanked everyone for the 5 years he had spent in the hospital. He was given the diagnosis of *dementia praecox*, probably because he had been severely disturbed for several years, and the cause of his illness was attributed to a ‘fear of being drafted into the Red Guards’. In his patient file, there is also a letter from the municipal authorities who, in 1942, had requested a medical certificate from Pitkäniemi to certify he was no longer mentally ill—Aleksanteri was going to get married. The hospital sent the necessary certificate stating he had been discharged in full health.^44^ This story thankfully had a happy end.

A similar case is that of 43-year-old Jaakko E., a tenant farmer and father of seven. In September, he was taken to Pitkäniemi by his sister-in-law, who told the psychiatrist on duty that Jaakko’s illness had started in February, when the war had broken out. As a socialist, Jaakko began to fear that he would be conscripted to the Red Guards and sent to the front line. To avoid the threat of conscription, he escaped into the woods and spent one night there. After returning home, he said he felt persecuted and was harbouring suicidal thoughts. Later on, he ran away again, and this time it took a week before he was found in a barn 5 km away from his house. He had buried himself so deep in a haystack that he could not get out of it by himself. His condition was very weak as he had not eaten or drunk anything all week. According to the local doctor’s referral, Jaakko had suffered from visual hallucinations in the woods, and was scared of falling into a trap, where he might get killed. In a certificate accompanying the referral, a local farmer and schoolteacher stated that he was actually afraid of getting arrested by the White Guards, because he was ‘known as a fierce socialist and an active Red’. So while Jaakko himself was saying that he was scared of the Reds, local White sympathisers gave a contrary statement, believing he had more reason to fear being arrested by the Whites.

In the ward, Jaakko ‘had a sad expression and was melancholic’. He was also paranoid that somebody would do away with him. His condition remained serious—in November 1920, he was reported to be lethargic, apathetic and sitting on his own with a blank stare on his face. In June 1921, he was not reacting to his environment and responded to questions with a few mumbling words. For an unknown reason, he was eventually discharged with an ‘unimproved’ condition and the diagnosis of *dementia praecox*. Fortunately however, like Aleksanteri, he recovered from his illness: in 1926, the municipal authorities informed the hospital that Jaakko was now in full health.^45^

Southern Tavastia was one of the few regions in Finland where the Red Guards tried to conscript soldiers; in most places they tried to recruit people voluntarily by paying them modest salaries. For Kustaa, Jaakko and Aleksanteri it also seemed that their condition was serious but transitory, as they all eventually recovered. It would be highly interesting to know just how many others there were like these three men in the war-torn country, especially in the regions controlled by the Reds, but also in the White areas. There were probably many men on both sides who were afraid of the frontline.

### The shock of killing.

The last fights of the Civil War took place in early May. Already in early April, after the Reds had lost the important city of Tampere, and German troops arrived in Finland to fight on the side of the Whites, it had become evident to both sides that the Whites would win the war. A few days after the war ended, 20-year-old Ilkka S. was admitted to Pitkäniemi. Son of a farmer and a volunteer serviceman in the White army, Ilkka had clear ‘hereditary burden’—two cousins on his father’s side were mentally ill. According to his father, he was quiet, unassuming and had ‘weak nerves’, but then tragedy hit when he started to see Reds everywhere while on the front line, and in a state of panic he began to shoot at people point-blank. One shot hit a fellow soldier in the head, and he died soon after. Ilkka was first taken to a military hospital in Vaasa, and then to his home, where he remained restless, gloomy and panicky. He was worried about the Reds entering his village, started suffering from auditory hallucinations, and then tried to harm his father. This was too much for his family, and he was taken to Pitkäniemi. In the ward, Ilkka was calm and compliant, and he showed no signs of mental illness. He was eventually discharged in February 1919, with his condition marked as having ‘improved’. He was given the diagnosis of *imbecillitas* (referring to moderate intellectual disability), which was less common among war patients; his other diagnosis was ‘mania’.^46^

The next two patients had been caught in the fierce battle between the Reds and Whites for Tampere, fought in late March and early April.^47^ This was the biggest battle yet witnessed in the Nordic countries, and resulted in the Whites taking the town and thousands of Reds prisoner.^48^ Two months later, in mid-June, Arvo T., the 21-year-old son of a farmer, was taken to Pitkäniemi. According to his mother, Arvo was a quiet, melancholic type and his mind was weak and slow. He had experienced learning difficulties in school, so he stopped studying and returned home to take up farming. When the Civil War broke out, he joined the White Guards, and after the fall of Tampere, he captured a Russian man who was trying to escape from the town. His superior officer ordered him to shoot the Russian right away, which he did.^49^ Several days later, after the chaos had subsided, Arvo lost his mind. He began to claim that all those who had shot Russians would in turn be shot, including himself. He would talk to himself, wriggle with pain and utter suicidal thoughts. The death of his father early that May only exacerbated his condition.

In the ward, Arvo was calm, compliant and melancholy. In July, he was working, and his conduct was quite normal. In late January 1919, in his unsent letter to his family, he wrote: ‘Life here is so dull and humdrum when you have to stay indoors all the time, and every night you are wrapped in those bloody sheets. Come and take me away, nobody wants to be here [...] I’m sure I’m in good health now’. But it seems the psychiatrists were of a different opinion, as he was kept in the hospital until August 1919, at which point he was discharged in full health and diagnosed with having suffered from ‘manic depressive insanity’.^50^

At the end of August, Juho K., the 19-year-old son of a shopkeeper, was taken to Pitkäniemi. His sister reported that, early in 1917, Juho had become mentally ill for 2 months. During this time, he was fearful and reluctant to go out of the house when he needed to run errands in another town. He also complained about losing his memory (and had suffered from bad hearing since catching scarlet fever at the age of three). After these 2 months, he returned to full health again until he went to see his relatives in Tampere in early April 1918. When he got there, it was after the Whites had invaded the town, and he was horrified to see dead bodies lying in the streets. When he returned home the following day, he became afraid of ‘evildoers’ and ‘shooters’, and he found himself closing doors in the house and jumping at small noises. In the certificate he wrote for Juho, the local doctor reported that Juho was expecting bad things to happen, had seen ghosts and other apparitions, and had mentioned suicide once—apparently thinking himself a bad person. He was also worried about his livelihood.

When he got to Pitkäniemi, Juho was melancholy, but his behaviour was organised. After three months, he recovered from his illness and admitted he had been so affected by the horrors he had witnessed in Tampere, that everything afterwards was ‘dark and gloomy’. In the ward, Juho was fully oriented and responsive, and he talked and behaved ‘politely’. In his patient record he was diagnosed as having manic-depressive insanity brought on by ‘seeing the effects of war’, and he was released ‘in full health’.^51^

Another patient shocked by the violent events of the war was Viljo P., the 25-year-old son of a tenant farmer. According to his brother, who took him to Pitkäniemi in March 1920, Viljo was conscripted into the White army in the spring of 1918, and what he witnessed during the war affected him deeply—most probably, Viljo had seen killing firsthand, or at least fought on the front and shot at the enemy. Before Christmas 1919, he stopped working and started to avoid others, occasionally talking in a confused manner. In Pitkäniemi, Viljo lay quietly in his bed and replied to questions slowly and in a quiet voice. He was melancholy and appeared gloomy, thinking himself as ‘weak’, but not insane. In December 1920, he committed suicide in the hospital. In his patient file he was diagnosed as having *dementia praecox*, but there are no details about this tragic event.^52^

It is impossible to say for sure whether Viljo’s suicide and his condition, which looks like a severe depression, was wholly attributable to his experiences during the Civil War. What is clear is that the war affected him deeply. It is quite possible that people like Viljo and Juho K., who had experienced atrocities first-hand during the Civil War, would today be diagnosed with post-traumatic stress disorder.

## The question of recovery

All the patients discussed in this article were deeply affected by the Civil War. This is most obvious in the case of the patients who were frightened of the Reds, of battles, and of the coming doom for the socialists. Before the war broke out, Anna T. was already starting to fear that the revolution would end badly for the Reds, while the other female patient cited here, Erika R., became frightened when the war was literally right outside her door. For Kaarlo J., the rural police officer, it was being chased by Reds through the woods which triggered his mental illness. The White serviceman Ilkka S. seems to have panicked and shot a fellow soldier accidentally, after which he had a nervous breakdown, while for another White, Arvo T., it was shooting a captured Russian after Tampere had been captured which caused him to flip. Anton H. feared the Reds who had killed two of his cousins and taken his brother to an unknown place, and Eino M. got scared when a Red patrol arrived in his house, because he knew they had already killed his brother. Equally, Kustaa L. became frightened when the Reds came to plunder the house he worked in as a farm hand. Juho K. was shocked by the sight of dead bodies lying in the streets of Tampere, and Viljo P. was also deeply affected by what he saw in the war after being conscripted to the White Guards—to the point where he committed suicide while at Pitkäniemi. Meanwhile, Aleksanteri K. was also conscripted, but to join the Red Guards, whom he feared. As for Jaakko E., a well-known socialist in his local community, he appeared to be afraid of both the Reds and Whites.

If we take into account all 24 patients admitted to Pitkäniemi for war-related reasons in 1918, we notice that exactly half were discharged in ‘full health’, three were ‘recovering’, six were discharged with an ‘unimproved’ condition, and three died in the hospital. The majority of patients had therefore either recovered or were recovering from mental illness when they were released. Interestingly, all those discharged as ‘unimproved’ were given the diagnosis of *dementia praecox*, while 10 out of the 12 patients in ‘full health’ had been diagnosed with manic-depressive insanity. The psychiatrists in Pitkäniemi were following the terminology for ‘functional psychoses’ used by the German psychiatrist, Emil Kraepelin. *Dementia praecox* was used for the patients they saw as chronically and seriously ill, while manic-depressive insanity covered those whose chances of recovery were considered to be relatively high. Of the three patients who died in the hospital, two had been given the diagnosis of dementia praecox, while the third most probably died as a result of physical abuse—the perpetrator was a male nurse.^53^ What is also worth noting is that eight of the 24 patients (from both fully recovered and unimproved categories) had members of their own family who had become mentally ill.

One must be careful when drawing conclusions based on 24 patients who were all treated in the same hospital. It is safe to say, however, that upon their release, the majority of these patients were seen as having recovered from their illness. Among the patients whose condition was ‘unimproved’, there was one who had suffered from severe symptoms of psychosis years before the Civil War. Another patient discharged as ‘unimproved’ was later reported to have fully recovered; this means there were only four patients who were still ill when they were released, and about whom we do not have any further information after they were discharged. It is of course possible that some of the patients who had supposedly recovered had a relapse later on, but at least we know they were not treated in Pitkäniemi again. This information suggests that, in the case of the Finnish Civil War, most patients admitted to mental hospitals suffered from an acute, reactive and transitory mental disorder.

In the case of the Finnish Civil War, the fact that it was a relatively short conflict may have helped recovery, especially of those individuals whose family members did not perish in the war itself or the prison camps afterwards. Then again, for those that did lose a loved one in the conflict, recovery must have been a slower and more painful process.

## The Impact of War on Other Mental Hospitals

In 1918, there were three state mental hospitals in Finland, and two of them were in the areas controlled by the Red Guards—Pitkäniemi near Tampere, and Lapinlahti in Helsinki. The third hospital—Niuvanniemi, near the eastern town of Kuopio—was in the area controlled by the Whites. In addition to these institutions, there were a few district and municipal mental hospitals.

The only psychiatrist killed by the Reds was Santeri Jalava, doctor for the town of Uusikaupunki in the south-west of the country, who was also in charge of the local district mental hospital. Both Jalava and the financial director of the hospital were arrested, and Jalava was killed by the side of the road, where the Red patrol left him after taking his winter coat. The probable reason he was shot was because he had been one of the founders of the White Protection Guard in Uusikaupunki in 1917.^54^ Although there is evidence that very few doctors were actually killed by the Reds, personal animosity towards the revolutionaries doubtlessly influenced the ways in which the insurgents, socialists and the working class as a whole were perceived by the academics and intellectuals after the war.

In 1918, the Niuvanniemi State Mental Hospital in Kuopio had 10 war patients—the specific reasons were ‘socialist uprising’, ‘imprisonment’ or ‘fear of military service’. The Turku Prison Mental Hospital also had 16 ‘criminal patients’ who had fought in the Red army. The following year (1919), the number of ‘Red’ patients in the hospital more than doubled (34 patients were Reds). Their crime was high treason, which was sometimes coupled with robbery or murder.^55^

In her study of the early history of the Kellokoski District Mental Hospital (located about 40 km north of Helsinki), Anna Riitta Jyrkinen has found 23 patients whose illness was related to the Civil War. Most patients were men, and most were diagnosed with manic-depressive insanity. According to Jyrkinen, all of them were frightened and were seeking asylum in the hospital. As many as 20 of them were discharged in the same year, which indicates that their suffering was a transitory mental reaction to the war. Jyrkinen observes that several of these 23 patients had relatives who had witnessed the killing or imprisonment of their near ones.^56^

When the war ended in May 1918, for the Reds the ordeal was anything but over. Between May and October, thousands of them were executed, and about 80,000 Reds—including people suspected of being on their side—were incarcerated in massive prison camps, where many died because of malnutrition, poor hygiene and infectious diseases. The legal historian Jukka Kekkonen has called the immediate period after the Civil War, when the rule of law descended into wanton revenge killing, ‘post-war cleansing’.^57^ In fact, the term ‘cleansing’ was used by the Whites themselves at the time.^58^ Altogether, nearly 30,000 Reds died either during or after the war, while the corresponding figure among the Whites was only 5,000. This means that the overwhelming majority of fatalities in the war—85 per cent—were Reds or sympathetic to the socialist revolution.

The squalid conditions of the prison camps affected the mental health of prisoners, who had no idea whether they would be executed, imprisoned for years or set free. It is unclear whether those displaying signs of mental disorder were taken to a hospital, but it is quite possible that some individuals whose mental illness was apparent to everyone were hospitalised. Among those admitted to Pitkäniemi Hospital, I have found two patients, both Reds, who had been prisoners of war, and it is a distinct possibility that more such patients were taken to the Prison Mental Hospital in Turku.

Most perpetrators on both sides were ordinary people, as were their victims. From the studies of Christopher Browning, Jan T. Gross, Jan Grabowski and others,^59^ we know that ordinary, law-abiding people can turn into ruthless killers when they are in situations where killing appears to be not only a viable option but the preferred solution, or at least something which, from their point of view, just needs to get done. It is fairly well-known that, until the late 1930s, it could be quite difficult to live in Finland as a socialist or former Red. What is less well-known is that the Whites did not always live that comfortably either, especially if they might have confronted individuals in their own communities who never forgave them for the ‘White repression’. In the archives of Oulu District Mental Hospital in Northern Finland, I have found the patient files of two working men who had been White Guards during the Civil War. In 1925 and 1930, respectively, they were harassed as traitors by their co-workers and others with sympathies for socialism. One of them spent four years in the hospital,^60^ and the other 10—the latter was diagnosed with neurosyphilis.^61^

For historians of mental health, one of the most striking things about the period directly after the Civil War was how the psychiatric significance of the war in Finland was almost completely ignored. This ‘professional silence’ could have been for a host of reasons; first of all, the pool of psychiatrists in the country was very small, and since nearly all of them worked in overcrowded and understaffed mental hospitals, they clearly made day-to-day clinical activities and treatment options their priority. This would also explain why most psychiatric publications in the 12-year period after the war (1918–30) deal with topical clinical issues. Second, the Finnish medical community, including psychiatrists, had more immediate health worries to deal with: the post-war pandemic of Spanish flu, *encephalitis lethargica* (sleeping sickness), neurosyphilis and cases of ‘feeblemindedness’—increasingly discussed within the framework of eugenics or ‘racial hygiene’.^62^

The third reason might also have been because the academic middle-class wanted to leave memories of the Civil War behind them so the scars of a divided nation could heal. Neither doctors, physicians, nor academics seemed able to dispassionately discuss what had shook their country directly after independence. Those articles and books published at the time about the war in general and the Red Guards in particular were by the winning side and understandably prone to bias. In many, psychiatric and psychological terms and theories were indeed used, but the authors’ purpose was not so much to discuss the effects of the civil war on the mental health of people, as to use psychopathological language to denigrate the Reds—and sometimes all socialists—by labelling them ‘psychotic’, ‘mentally deranged’ or as hapless victims of ‘mass suggestion’. The then-popular ideas of Gustave Le Bon’s crowd psychology were frequently employed to make the case that the ‘socialist masses’ had been driven into a frenzy, where they internalised the commands and promises of their Red leaders who, for their part, were willing and able to manipulate the unconscious impulses of their followers and channel their energies to their own political ends.^63^

## Conclusion

After the nightmare of 1918, Finland was reborn as a nation. First in October, and then in December 1918, the head of state pardoned nearly 17,000 Red prisoners, and, after local elections, the city council of war-torn Tampere had a Social Democratic majority. In the parliamentary elections the following spring, the Social Democratic Party also became the biggest party in Parliament. In effect, this meant that the policy of repression was eased with the re-emergence of the labour movement in the public sphere. Finland became by constitution a parliamentary democracy, and within a few years Parliament enacted a land reform, a school reform, the 8-h working day, and a tax reform.^64^ A nation that had started out as a textbook example of a failed state was transformed into a relatively stable ‘republic of farmers and workers’ in the space of a few years. Yet, political tensions were not easily resolved. For example, the majority of Red ex-prisoners did not enjoy full citizens’ rights until the 1920s—they were only given parole, which meant they were not eligible to vote or be in public office. Furthermore, there continued to be politically motivated trials in which communists were given prison sentences for making plans to commit high treason. These political trials only ended in 1939 when the war with the Soviet Union began and changes to the judicial system pushed these trials into the background. Nevertheless, between 1918 and 1939, altogether 4,000 sentences were handed out for treason or high treason.^65^

Despite the obviously traumatising effects of the Civil War on the nation as a whole, individuals who went through these shocking experiences appeared to be quite resilient. I would suggest that, even in a civil war, most mental disturbances could be reactive and often, but not always, transient. To fall ill because of a frightening experience is a serious, yet natural emotional (and physiological) reaction to the horrors of war. As my discussion of the patient cases in this article indicates, with time the reactive symptoms of patients became milder and their mental health returned insofar as, once released from hospital, they could carry on with their everyday life in their community. I am not making the preposterous claim that war-related traumas become completely healed with time, but I do claim that in a sufficiently secure and supportive environment, humans suffering from reactive mental disorders can regain their mental health and become socially more or less competent. Studies conducted on psychological resilience, including the resilience of Holocaust survivors, would appear to lend support to this suggestion.^66^

To recover from an acute episode of mental illness is not the same as to regain full health, or to lead a happy and fulfilling life. What happened in Finland after the Civil War was that the continuing agony of the Reds—and, by extension, the working class—left an open wound in the nation’s ‘soul’, and it also ensured that the victors could not simply leave the bloody past behind and look forward to a harmonious, conflict-free future for the young democracy. What had once been a republic of fear and hate could not be turned into a republic of hope and harmony as long as a large segment of society were treated as second-class citizens. Therefore, even though the nation recovered from the madness of the Civil War quite rapidly, it continued to suffer from a low-grade inflammation in its body politic. At the individual level, in the homes, workplaces and schools across the country, memories of the horrors of war haunted people for years to come. In this regard, the civil wars in Finland and Spain have distinct similarities: as in Finland, so in Spain the detrimental psychological effects of civil war haunted the nation for decades. Also, in both countries the question of war trauma was not discussed by the mental health professionals in the post-war era.^67^ In Finland, this question became unavoidable only during World War II, when thousands of traumatised soldiers were sent to mental hospitals to receive treatment.^68^

Finally, even though a comparative study of mental suffering due to civil war might not reveal striking similarities between civil wars in Europe (or the world), it would nevertheless be a worthwhile effort to examine these conflicts from the transnational perspective of the history of emotions. Such a study might bring new knowledge of, and insight into, the ways in which emotions are mobilised and sustained in nations shattered by civil war.

## Footnotes

1 For book-length studies of the Civil War, see Tuomas Tepora and Aapo Roselius, eds, *The Finnish Civil War 1918: History, Memory, Legacy* (Leiden: Brill, 2018); Anthony F. Upton, *The Finnish Revolution 1917-1918* (Minneapolis: University of Minnesota Press, 1980); Risto Alapuro, *State and Revolution in Finland* (Leiden: Brill, 2019); Tuomas Hoppu and Pertti Haapala, eds, *Tampere 1918: A Town in the Civil War* (Tampere: Vapriikki, 2010). See also Juha Siltala, ‘Dissolution and Reintegration in Finland, 1914–1932: How Did a Disarmed Country Become Absorbed into Brutalization?’ *Journal of Baltic Studies*, 2015, 46, 11–33; Juha Siltala, ‘National Rebirth Out of Young Blood. Sacrificial Fantasies in the Finnish Civil War, 1917–1918’. *Scandinavian Journal of History*, 2006, 31, 290–307.

2 On the history of civil wars in Europe during the first half of the twentieth century, see Stanley G. Payne, *Civil War in Europe, 1905-1949* (Cambridge: Cambridge University Press, 2011).

3 Julián Casanova, ‘Civil Wars, Revolutions and Counterrevolutions in Finland, Spain, and Greece (1918-1949): A Comparative Analysis’. *International Journal of Politics, Culture, and Society*, 2000, 13, 515–537; Payne, *Civil War in Europe*, 25–32.

4 For a list of situational factors influencing the escalation of the Civil War, see Siltala, ‘Dissolution and Reintegration’, 27–29.

5 Aapo Roselius, ‘The War of Liberation, the Civil Guards and the Veterans’ Union’, in Tepora and Roselius, eds, *The Finnish Civil War*, 312.

6 Broadly speaking, the main difference between the Reds and the Whites was that the former were workers and other non-owners, and the latter were (more or less) middle-class owners of property (land, capital, resources). Pertti Haapala, ‘The Expected and Non-expected Roots of Chaos: Preconditions of the Finnish Civil War’, in Tepora and Roselius, eds, *The Finnish Civil War*, 31.

7 For literature on war traumas and war psychiatry, useful recent publications to consult are Jason Crouthamel and Peter Leese, eds, *Psychological Trauma and the Legacies of the First World War* (Palgrave Macmillan, 2017); Jason Crouthamel and Peter Leese, eds, *Traumatic Memories of the Second World War and After* (Palgrave Macmillan, 2016); and Livia Prüll and Philipp Rauh, eds, *Krieg und medikale Kultur. Patientenschicksale und ärztliches Handeln in der Zeit der Weltkriege 1914-1945* (Göttingen: Wallstein, 2014). Regarding Finland in World War II, see Ville Kivimäki, *Battled Nerves: Finnish Soldiers’ War Experience, Trauma, and Military Psychiatry, 1941–44* (Turku: Åbo Akademi University, 2013).

8 For the Spanish Civil War which in some ways, is comparable to the Finnish Civil War, see Stephanie Wright, ‘My Husband … is an Authentic Psychopath’: Spanish Civil War Veterans, Mental Illness and the Francoist Regime. *Social History of Medicine*, 2021, 34, 1236–1255; Olga Villasante, ‘“War neurosis” during the Spanish Civil War (1936-39)’. *History of Psychiatry*, 2010, 21, 424–435; Paloma Vázquez de la Torre and Olga Villasante, ‘Psychiatric Care at a National Mental Institution During the Spanish Civil War (1936-39): Santa Isabel de Leganés’. *History of Psychiatry*, 2016, 27, 51–64; Rafael Huertas, ‘Spanish Psychiatry: The Second Republic, the Civil War, and the Aftermath’. *International Journal of Mental Health*, 2006, 35, 54–65; for the Russian Civil War, see Sean Guillory, ‘The Shattered Self of Komsomol Civil War Memoirs’. *Slavic Review*, 2012, 71, 546–565.

9 Adam Smith, *Theory of Moral Sentiments* (New York: Prometheus Books, 2000), 8.

10 Jan Plamper and Benjamin Lazier, eds, *Fear: Across the Disciplines* (Pittsburgh: University of Pittsburgh Press, 2012), 1

11 Jan Plamper, ‘Soldiers and emotion in early twentieth-century Russian military psychology’. In Plamper and Lazier, eds, *Fear*, 78–98.

12 Michal Shapira, *The War Inside: Psychoanalysis, Total War, and the Making of the Democratic Self in Postwar Britain* (Cambridge: Cambridge University Press, 2013), 32.

13 Joanna Bourke, ‘Fear and Anxiety: Writing about Emotion in Modern History’. *History Workshop Journal*, 2003, No. 55, 111–133, 124; Ute Frevert, ‘The Modern History of Emotions: a Research Center in Berlin’. *Cuadernos de Historia Contemporánea* 2014, 36, 31–55, 39. For more on moral emotions, see Jonathan Haidt, ‘The Moral Emotions’. In R. J. Davidson, K. R. Scherer, abd H. H. Goldsmith, eds, *Handbook of Affective Sciences* (Oxford: Oxford University Press, 2003), 852–870.

14 Edgar Jones, ‘The Psychology of Killing: The Combat Experience of British Soldiers during the First World War’. *Journal of Contemporary History*, 2006, 41, 229–246; 231.

15 As discussed later in this article, it is easy to find psychological interpretations of the Civil War and the Reds in numerous treatises published in the years immediately following the war. In the patient records, however, I have not seen any references made to the then popular theories of male hysteria, shell shock and constitution.

16 Shapira, *The War Inside*, 27.

17 Anders Ahlbäck, ‘Masculinities and the Ideal Warrior: Images of the Jäger Movement’, in Tepora and Roselius, eds, *The Finnish Civil War*, 258; Siltala, ‘Dissolution and Reintegration’, 20.

18 As the historian Tuomas Hoppu has pointed out, ‘it was fairly common that the Red Guard would send men with hardly any military training to the front only a few days after they had joined up’. Tuomas Hoppu, ‘Front-line Battles’, in Hoppu and Haapala, eds, *Tampere 1918*, 71.

19 On the history of the psychiatric interest in heredity, see Theodore M. Porter, *Genetics in the Madhouse: The Unknown History of Human Heredity* (Princeton: Princeton University Press, 2018).

20 In addition to psychiatrists, neurologists, internists and other representatives of somatic medicine took into account psychological symptoms and their possible hereditary nature. See for example, Armas Cederberg, ‘Trauma ja sydänviat’. *Duodecim* 1917, 33, 321–335.

21 Karl Kleist, ‘Schreckpsychosen’. *Allgemeine Zeitschrift für Psychiatrie und psychiatrisch-gerichtliche Medizin* 1918, 74, 171–172, 432–510.

22 Corey Robin, *Fear: The History of a Political Idea* (Oxford: Oxford University Press, 2004), 43, 49.

23 Uno Harva, *Suomalaisten muinaisusko* (Porvoo: WSOY, 1948), 246–261; Risto Pulkkinen, *Suomalainen kansanusko samaaneista saunatonttuihin* (Helsinki: Gaudeamus, 2014), 143–147.

24 Ulla-Maija Peltonen, ‘Civil War Victims and the Ways of Mourning in Finland in 1918’, in Kenneth Christie and Robert Cribb, eds, *Historical Injustice and Democratic Transition in Eastern Asia and Northern Europe* (New York: Routledge Curzon, 2002), 184–197; Mandy Lehto, ‘Remembering the Finnish Civil War’, in Christie and Cribb, eds, *Historical Injustice*, 198–209.

25 On the relations between the labour movement and Church, see Tuomas Tepora, ‘The Mystified War: Regeneration and Sacrifice’, in Tepora and Roselius, eds, *The Finnish Civil War*, 159–200, 173–174; Huhta, *Sisällissota ja kirkko*.

26 Viljo Sohkanen, *Punakaartilaisen päiväkirja* (Joensuu: Kirjokansi, 2017), 70. Translation by the author of this article.

27 Mervi Kaarninen and Pekka Kaarninen, *Pitkäniemen sairaala 1900-1990* (Tampere: Pitkäniemen sairaalan kuntainliitto, 1990), 86–87.

28 Petteri Pietikäinen, *Kipeät sielut. Hulluuden historia Suomessa* (Helsinki: Gaudeamus, 2020).

29 Suomen Virallinen Tilasto XI:35, 1918, 76.

30
*Ibid*.

31 These patient records contain sensitive personal data, and in my analysis of these records I have tried to do justice, on the one hand, to the patients as well as to the medical professionals in hospitals, and, on the other hand, to truthfulness. Specifically, I have tried to follow the ‘epistemic virtues’ as expounded by Jeroen van Dongen and Herman Paul in the book they have edited (*Epistemic Virtues in the Sciences and the Humanities*. Leiden: Brill, 2017). These virtues refer to truthfulness, impartiality, sincerity and accuracy. On the last two (accuracy and sincerity) see also Bernard Williams, *Truth and Truthfulness: An Essay in Genealogy* (Princeton: Princeton University Press, 2004).

32 For more on the White and Red Terror, see Marko Tikka, ‘Warfare and Terror in 1918’, in Tepora and Roselius, eds, *The Finnish Civil War*, 90–118.

33 Pitkäniemi Hospital Archive 3206/18.

34 Pietikäinen, *Kipeät sielut*, 64–65, 217.

35 On the Lutheran Church during the Civil War, see Ilkka Huhta, ed, *Sisällissota ja kirkko* (Helsinki: Suomen Kirkkohistoriallinen Seura, 2009).

36 All the names of patients in this article have been changed.

37 For more on the ‘Suinula Massacre’, see Tuomas Hoppu, ‘Front-line Battles’, in Hoppu and Haapala, eds, *Tampere 1918*, 58–59; Marianne Junila, ‘War Through the Children’s Eyes’, in Tepora and Roselius, eds, *The Finnish Civil War*, 237.

38 The National Archives, Hämeenlinna, Pitkäniemi Hospital Archives 1900-1954, Patient records, 3185/18 (henceforth, Pitkäniemi Hospital Archive).

39 Pitkäniemi Hospital Archive 3189/18.

40 Pitkäniemi Hospital Archive 3191/18.

41 On the use of work as therapy in international psychiatry, see Waltraud Ernst, ed, *Work, Psychiatry and Society, c. 1750–2015* (Manchester: Manchester University Press, 2015). On work therapy in Finnish mental hospitals, see Anu Rissanen, ‘Treatment and Rehabilitation: Patients at Work in Finnish Mental Institutes’, in Tuomas Laine-Frigren, Jari Eilola and Markku Hokkanen, eds, *Encountering Crises of the Mind: Madness, Culture and Society, 1200s–1900s* (Leiden: Brill, 2018), 196–221.

42 Pitkäniemi Hospital Archive 3223/18.

43 Pitkäniemi Hospital Archive 3225/18.

44 Pitkäniemi Hospital Archive 3229/18.

45 Pitkäniemi Hospital Archive 3249/18.

46 Pitkäniemi Hospital Archive 3211/18.

47 See Hoppu and Haapala, *Tampere 1918*.

48 On the experiences of schoolgirls living in Tampere during the war, see Junila, ‘War Through the Children’s Eyes’, 230–253.

49 On the executions of Russians in the Finnish Civil War, see Jyrki Loima, ‘Genocide and Ethnic Cleansing? The Fate of Russian “Aliens and Enemies” in the Finnish Civil War in 1918’. *The Historian*, 2007, 69, 254–274.

50 Pitkäniemi Hospital Archive 3226/18.

51 Pitkäniemi Hospital Archive 3247/18.

52 Pitkäniemi Hospital Archive 3456/20.

53 Pitkäniemi Hospital Archive 1918/3246.

54 Mikko Uola, *Lahtarin hengen hinta* (Mikko Uola [private publisher]: Keuruu, 2019), 84–86.

55 Antti Parpola, *Toivo/häpeä: Psykiatria modernissa Suomessa* (Helsinki: Suomen psykiatriyhdistys, 2013), 58.

56 Anna Riitta Jyrkinen, *Kellokosken piirimielisairashoitolassa vuosina 1915-1918* (Kuopio: Kuopion yliopisto, 2005), 103–110.

57 Jukka Kekkonen, *Kun aseet puhuvat: Poliittinen väkivalta Espanjan ja Suomen sisällissodissa* (Helsinki: Art House, 2016), 345.

58 Aapo Roselius, *Teloittajien jäljillä* (Helsinki: Tammi, 2006), 12–13.

59 Christopher R. Browning, *Ordinary Men. Reserve Police Battalion 101 and the Final Solution in Poland* (New York: Harper Perennial, 1998); Jan T. Gross, *Neighbors: The Destruction of the Jewish Community in Jedwabne, Poland* (Princeton: Princeton University Press, 2001); Jan Grabowski, *Hunt for the Jews: Betrayal and Murder in German-Occupied Poland* (Bloomington: Indiana University Press, 2013).

60 Northern Ostrobothnia Hospital District, Oulu District Mental Hospital archive, Patient records, 73/25.

61 Northern Ostrobothnia Hospital District, Oulu District Mental Hospital archive, Patient records, 230/30.

62 On the history of racial hygiene in Finland, see Markku Mattila, *Kansamme parhaaksi: rotuhygienia Suomessa vuoden 1935 sterilointilakiin asti* (Helsinki: Suomen historiallinen seura, 1999), Summary: ‘In our nation’s best interest: eugenics in Finland until the promulgation of the sterilization law of 1935’.

63 On the application of crowd psychology to the discussions of the civil war and the Red Guards, see Petteri Pietikainen, ‘”A psychological riddle demanding a solution”. Crowd psychology and the Finnish Civil War of 1918.’ *History of European Ideas* 2022, 48, 555–573.

64 Tuomas Forsberg, ‘Post-Conflict Justice and the Finnish Civil War 1918’. *Finnish Yearbook of International Law*, 2007, 18, 137–154.

65 Lars Björne, *“…syihin ja lakiin eikä mielivaltaan…” Tutkimus Turun hovioikeuden poliittisista oikeudenkäynneistä vuosina 1918-1939* ([Helsinki]: Suomalainen lakimiesyhdistys, 1977). In 1930, a law forbidding communist activities, including press, came into force. It was rejected in 1934, but the far left continued to be repressed until the end of the 1930s.

66 Peter Suedfeld, *Life After the Ashes. The Postwar Pain, and Resilience of Young Holocaust Survivors* (Washington: United States Holocaust Memorial Museum, 2002). On resilience, see also John W. Reich, Alex J. Zautra and John Stuart Hall, eds, *Handbook of Adult Resilience* (New York: The Guildford Press, 2012), 213–237; Michael Ungar, ed, *The Social Ecology of Resilience: A Handbook of Theory and Practice* (New York: Springer, 2013), 369–386; Michael Ungar, ‘Resilience Across Cultures’. *The British Journal of Social Work* 2008, 38, 218–235; Linda Liebenberg and Michael Ungar, *Researching Resilience* (Toronto: University of Toronto Press, 2009).

67 On Spanish Civil War veterans and mental illness, see Wright, ‘My Husband … is an Authentic Psychopath’.

68 Kivimäki, *Battled Nerves*.

